# HMMPred: Accurate Prediction of DNA-Binding Proteins Based on HMM Profiles and XGBoost Feature Selection

**DOI:** 10.1155/2020/1384749

**Published:** 2020-03-28

**Authors:** Xiuzhi Sang, Wanyue Xiao, Huiwen Zheng, Yang Yang, Taigang Liu

**Affiliations:** ^1^College of Information, Shanghai Ocean University, Shanghai 201306, China; ^2^School of Information, Syracuse University, Syracuse, NY 13244, USA; ^3^School of Engineering, University of Melbourne, Victoria 3010, Australia; ^4^School of Information Management, Nanjing University, Nanjing 210023, China

## Abstract

Prediction of DNA-binding proteins (DBPs) has become a popular research topic in protein science due to its crucial role in all aspects of biological activities. Even though considerable efforts have been devoted to developing powerful computational methods to solve this problem, it is still a challenging task in the field of bioinformatics. A hidden Markov model (HMM) profile has been proved to provide important clues for improving the prediction performance of DBPs. In this paper, we propose a method, called HMMPred, which extracts the features of amino acid composition and auto- and cross-covariance transformation from the HMM profiles, to help train a machine learning model for identification of DBPs. Then, a feature selection technique is performed based on the extreme gradient boosting (XGBoost) algorithm. Finally, the selected optimal features are fed into a support vector machine (SVM) classifier to predict DBPs. The experimental results tested on two benchmark datasets show that the proposed method is superior to most of the existing methods and could serve as an alternative tool to identify DBPs.

## 1. Introduction

DNA-binding proteins (DBPs), which can bind to and interact with DNA, play prominent roles in the structural composition of DNA and the regulation of genes. These proteins have a variety of biochemical functions in the cell and molecular biology, including the participation and regulation of various cellular processes, such as transcription, DNA replication, recombination, modification, and repair [1, 2]. Besides, DBPs are key components of steroids, antibiotics, and cancer drugs in the pharmaceutical industry [3]. Hence, the prediction of DBPs has become one of the research focuses in the field of protein science due to its significance in the related biological activities. In early studies, DBPs were normally identified by experimental techniques, such as filter binding assays, genetic analysis, X-ray crystallography, ChIP-chip analysis, and nuclear magnetic resonance (NMR) [4]. However, conventional experimental methods are often time-consuming and laborious. With the rapid increase of protein sequence data, there is a great need to develop efficient computational methods to identify DBPs solely based on their primary sequences.

From the machine learning perspective, identification of DBPs is usually considered a binary classification problem. In recent years, many computational methods have been applied to solve this problem. These methods primarily focus on the following two aspects: (1) the construction of encoding schemes for protein sequences and (2) the application of classification algorithms. Many machine learning techniques have been adopted to perform the prediction of DBPs, including support vector machine (SVM) [5–7], random forest (RF) [8–10], naive Bayes classifier [4], ensemble classifiers [11–13], and deep learning [14–16]. Among these algorithms, SVM and RF have been widely used because of their excellent performance. The existing SVM-based predictive methods differ in encoding schemes for protein sequences. A great number of sequence features have been applied to represent protein sequences into fixed-length numeric vectors, such as amino acid composition (AAC) [17], dipeptide composition [18], pseudo-AAC [19–22], position-specific score matrix (PSSM) profile [23–27], predicted secondary structure [28], and hidden Markov model (HMM) profile [29].

Numerous researches have proved that evolutionary information encoded in the PSSM profile is more informative than protein sequence alone [30]. The PSSM profiles have been widely used in bioinformatics, such as protein remote homology detection [31], protein fold recognition [32], and prediction of protein structural class [33]. Accordingly, PSSM-based feature descriptors have successfully enhanced the prediction accuracy of DBPs. For example, Kumar et al. [24] first adopted the PSSM profile to identify DBPs and constructed an SVM model called DNAbinder. Waris et al. [25] further developed a classifier by integrating the PSSM profile and other two protein representations, i.e., dipeptide composition and split AAC. Besides, the method of Wang et al. [26] applied the discrete cosine transform and the discrete wavelet transform to compress the PSSM profile and achieved excellent prediction performance. Wei et al. [9] proposed a powerful predictor called Local-DPP, which combined the local pseudo-PSSM features with the RF classifier. Recently, Zaman et al. [29] build a predictive model based on the HMM profile instead of the PSSM profile for the detection of DBPs and experimentally showed the effectiveness of the HMM-based features by using the jackknife test on the benchmark dataset. However, the method proposed by Zaman et al. performed relatively poorly on the independent dataset test [29]. It appears that evolutionary information in the form of HMM profile has not been adequately explored and there is still room for developing more effective feature extraction techniques to improve the prediction performance of DBPs.

To this end, we propose a novel method, called HMMPred, which utilizes features extracted solely from the HMM profile to further improve the prediction accuracy of DBPs. First, HMM profiles are transformed into fixed-length feature vectors with the joint use of three feature extraction methods including AAC, auto covariance transformation (ACT), and cross-covariance transformation (CCT). Next, the extreme gradient boosting (XGBoost) algorithm is adopted as a feature selection technique to pick the well-distinguished features. Finally, these selected optimal features are fed into an SVM classifier to make predictions. Validation results on two working datasets indicate that the proposed method performs better than most of the other existing predictors, especially the remarkably high accuracy on the independent dataset.

## 2. Materials and Methods

**Figure 1:**
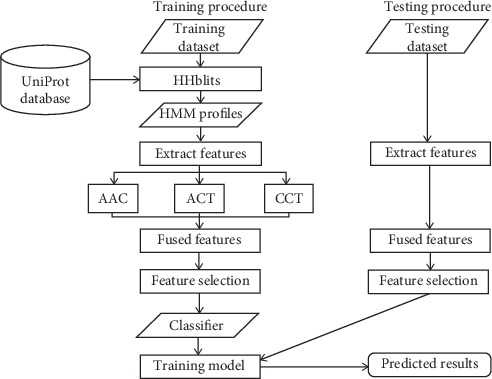
Framework of the proposed method for DBPs prediction.

This section illustrates all details about our proposed method and the following flow chart (Figure 1) clearly presents the process framework of the method. This process involves both training and testing stages. For the training phase, the HMM profiles of query proteins are generated by running the HHblits program, which is an effective sequence alignment tool with less running time but higher sensitivity and accuracy than PSI-BLAST [34]. Next, features are extracted from the HMM profiles by fusing three techniques, i.e., AAC, ACT, and CCT. Then, the optimal features are selected and finally inputted into a classifier for the subsequent model training and DBPs prediction. For the testing phase, a series of procedures are similar to those in the previous part so that the prediction result can be obtained after feeding selected features into the training model, which is generated in the training stage.

### 2.1. Datasets

Two benchmark datasets, PDB1075 [22] and PDB186 [4], are used to measure the performance of the proposed method. The PDB1075 dataset which contains 525 DBPs and 550 non-DBPs is first applied for model training as well as testing by adopting cross-validation (CV) methods. On the other hand, the PDB186 dataset is adopted for an independent test to further evaluate the robustness and generalization ability of our predictor, which includes 93 DBPs and 93 non-DBPs. These protein sequences in the two datasets are selected from the Protein Data Bank [35] through a rigorous filtering procedure: (1) remove the sequences with a length of less than 50 amino acids or unknown residues such as “X”; and (2) cut off those sequences that have more than 25% sequence similarity with any other sequences.

### 2.2. Protein Sequence Representation

#### 2.2.1. HMM Profiles

A previous study has shown that HMM profiles are more effective for DBPs prediction compared with PSSM profiles [29]. In this study, the HMM profile is generated by performing four iterations of HHblits against the newest UniProt database [36] with an *E*-value threshold of 0.001. Given a query protein of length *L*, the size of HMM profile is *L* × 30. The values in HMM profile are converted to the range of (0, 1) by using the function *f*(*x*) = 2^−*x*/1000^, where *x* is the original HMM value. Similar to the PSSM profile, we only use the first 20 columns of HMM profile.

#### 2.2.2. Feature Extraction from HMM Profiles

Three feature extraction methods, i.e., AAC, ACT, and CCT, are adopted to transform HMM profiles into fixed-length feature vectors. It is well known that DNA-binding preference of a protein is closely related to its AAC features [17]. To compute AAC features from the HMM profile, the following formula is used:

(1) $$\begin{array}{c} \bar{h_{j}}=\frac{1}{L}\overset{L}{\underset{i=1}{\sum }}h_{i,j}\;\left(j=1,2,\cdots ,20\right), \end{array}$$

where *L* is the length of the protein sequence and *h*_*i*,*j*_ represents the element at the *i*^th^ row and *j*^th^ column of the HMM profile. In this way, 20 AAC features are obtained in total.

Obviously, if only AAC features are used to represent the protein, all the sequence-order information would be lost. To solve this problem, we apply ACT and CCT to reflect the local sequence-order effect. These two techniques have been widely used to extract features from the PSSM profile [37–39]. Thus, in this work, ACT and CCT are also adopted to convert the HMM profile into two numerical vectors by using the following equations:

(2) $$\begin{array}{c} A_{j,g}=\frac{1}{L-g}\overset{L-g}{\underset{i=1}{\sum }}\left(h_{i,j}-\bar{h_{j}}\right)\left(h_{i+g,j}-\bar{h_{j}}\right),\\ C_{j,k,g}=\frac{1}{L-g}\overset{L-g}{\underset{i=1}{\sum }}\left(h_{i,j}-\bar{h_{j}}\right)\left(h_{i+g,k}-\bar{h_{k}}\right), \end{array}$$

where 1 ≤ *j*, *k* ≤ 20, *j* ≠ *k*, and *g* is the lag. Hence, the number of ACT features is 20 × *G*, and the number of CCT features is 20 × 19 × *G* = 380 × *G*, where *G* is the maximum of *g*. As a result, each protein sequence can be represented as a (20 + 400 × *G*)-dimensional vector by fusing the AAC, ACT, and CCT features.

### 2.3. Feature Selection Algorithm

Feature selection plays a vital role in machine learning and pattern recognition, which can improve the performance of prediction models by removing irrelevant, noisy, and redundant information from the untreated features. In this study, we first obtain feature importance scores by applying RF and XGBoost algorithms individually. In the RF strategy, the importance of features is calculated by a total decrease in tree-node impurities from splitting off the predictor feature variable and is averaged over all sub-trees [40, 41]. The XGBoost method calculates an importance score for each feature based on its participation in making key decisions with boosted decision trees as suggested in [42]. Then, all of the features are ranked according to their importance scores. Finally, we select an optimal feature subset based on the ranked features. To the best of our knowledge, the XGBoost feature selection technique has not been explored for DBPs prediction.

### 2.4. Classification Algorithm

Two robust machine learning techniques, i.e., SVM and RF, are applied to perform the prediction of DBPs, which have been widely used for many classification tasks in the field of computational biology [43–46]. SVM is an outstanding classification method that is used to deal with a binary pattern recognition problem [47]. Its core idea is to find an optimal hyperplane as a decision surface, by maximizing the margin of separation between the two classes in the data. With the help of kernel tricks, SVM not only can classify the linearly separable samples but also can handle classes with complex nonlinear decision boundaries. Popular kernels used with SVMs include linear, polynomial, sigmoid, and radial basis function (RBF). In this study, the RBF kernel is adopted due to its excellent performance in the previous tests and the values of parameters *C* and *γ* are optimized between 2^−10^ and 2^10^ based on the 10-fold CV using a grid search strategy.

RF, as an ensemble learning algorithm, is not only widely used in feature selection which is discussed before but also applied in classification [48]. It is composed of many decision trees, and each tree in the forest makes a judgment on the sample to determine whether it belongs to positive instances or negative ones. Then, all voting results from each tree are collected to finally classify the samples into the category with the maximum votes. The SVM and RF algorithms were implemented using the Python sklearn library [49]. All experiments in this study were carried out in version 3.7 of Python.

### 2.5. Performance Evaluation

The performance of HMMPred is evaluated by three commonly used tests: 10-fold CV and jackknife CV implemented on the PDB1075 dataset, and an independent test where the PDB1075 dataset is used to train the model and testing is on the PDB186 dataset. All results are reported using the following four performance metrics: sensitivity (SN), specificity (SP), accuracy (ACC), and Matthew's correlation coefficient (MCC) [50, 51]. These metrics are formulated as follows:

(3) $$\begin{array}{c} \text{SN}=\frac{\text{TP}}{\text{TP}+\text{FN}},\\ \text{SP}=\frac{\text{TN}}{\text{TN}+\text{FP}},\\ \text{ACC}=\frac{\text{TP}+\text{TN}}{\text{TP}+\text{FP}+\text{TN}+\text{FN}},\\ \text{MCC}=\frac{\text{TP}\times \text{TN}‐\text{FP}\times \text{FN}}{\sqrt{\left(\text{TP}+\text{FN}\right)\left(\text{TP}+\text{FP}\right)\left(\text{TN}+\text{FP}\right)\left(\text{TN}+\text{FN}\right)}}, \end{array}$$

where TN, FN, TP, FP, respectively, represent the number of true negative, false negative, true positive, and false positive samples predicted. In addition, we also compute the area under the receiver operating characteristic (ROC) curve (AUC), which is a preferred metric for evaluating the performance of a binary classifier.

## 3. Results and Discussion

### 3.1. The Impact of the Parameter *g* on Prediction Performance

**Figure 2:**
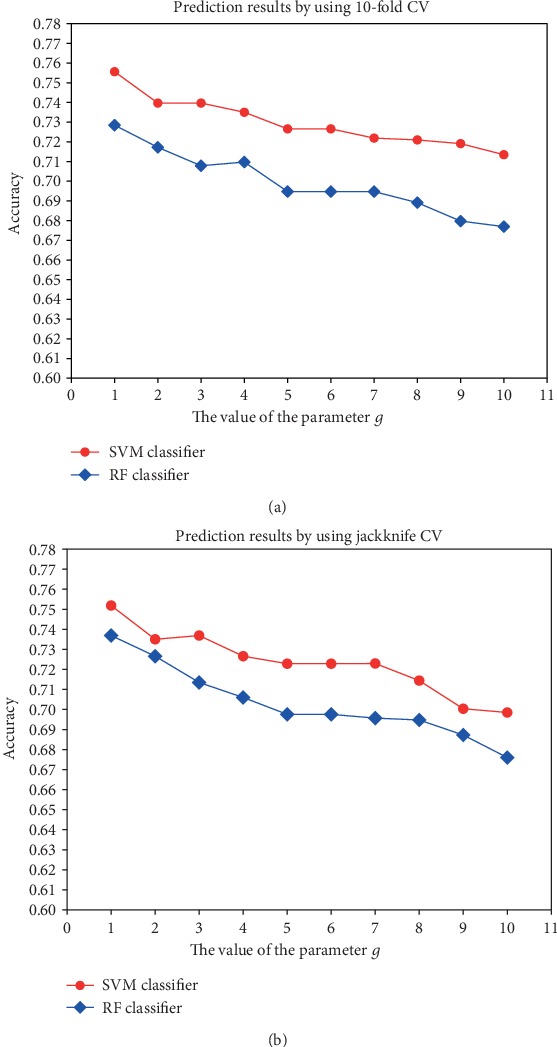
This shows how different *g* values affect the accuracies based on two CV methods. (a) The prediction results by using the 10-fold CV method. (b) The prediction results by using the jackknife CV method.

The ACT and CCT features represent the average correlation of two amino acids separated by *g* positions along the query protein sequence. To investigate the impact of parameter *g* on the prediction performance, we compare the prediction results by increasing the value of *g* from 1 to 10 with an increment value of 1, using the RF classifier and the SVM classifier under two different evaluation methods individually. Given that the accuracy rate is used as a crucial evaluation criterion in the model assessment (Figure 2), some insights into the selection of optimal *g* value and classifier are summarized below.

The following figures exhibit two striking traits. Firstly, the accuracy rate dwindles with the gradual increases of parameter *g*. Secondly, the accuracies of the SVM classifier are consistently better than those of the RF classifier. Referring to Figure 2(a), when the value of *g* is greater than 7, both SVM and RF classifiers show relatively poor performance. In addition, the accuracies remain relatively stable with *g* ranging from 5 to 7. A similar conclusion could be drawn from Figure 2(b). On the other hand, the increment of *G* (i.e., the maximum of *g*) followed by the growth of feature dimension could cause issues of feature redundancy, additional computational cost, and extra time consumption. Hence, to make a trade-off between the accuracy rate and the number of feature dimension, keeping the maximum of *g* to 5 is recommended. Accordingly, the number of ACT features is 100 and the number of CCT features is 1900.

### 3.2. Comparative Analysis of Different Classifiers with Different CV Methods

In this section, we further compare the performance between SVM and RF classifiers combined with four different feature extraction techniques including AAC, ACT, CCT, and AAC+ACT+CCT, respectively. Results on the PDB1075 dataset by using two CV tests are listed in Tables 1 and 2.

As shown in Table 1, the combination of SVM classifier with AAC+CCT+CCT features achieves the highest accuracy rate (0.8034) compared with others using the same classifier but with different features. Both MCC and AUC measures also give similar results. Meanwhile, the AAC feature and CCT feature obtain the highest SN and SP, respectively, suggesting that these two features are crucial to the identification of DBPs. For the RF classifier, AAC+ACT+CCT is also deemed to be the most appropriate method. Except for SP and AUC, the results of AAC+ACT+CCT consistently outperform the other three feature extraction methods. Apparently, the SVM classifier is more superior to the RF classifier in this experiment.

According to Table 2, similar conclusions can be reached by using the jackknife CV. For the SVM classifier, AAC+ACT+CCT is considered the optimal method with an accuracy rate of 0.8015. The RF classifier provides the accuracy rate of 0.7706 by using AAC+ACT+CCT features, which is higher than the cases with ACT and CCT features but is lower than the case with AAC features (0.7930). This suggests that multifeature fusion could generate irrelevant noise information and feature selection is necessary to enhance the prediction of DBPs in the next step.

Therefore, after analysing the data obtained from the examinations above, the combination of the SVM classifier with the joint use of the AAC+ACT+CCT features is adopted in the subsequent analysis due to its finest achievement.

### 3.3. Performance Analysis of Feature Selection

**Figure 3:**
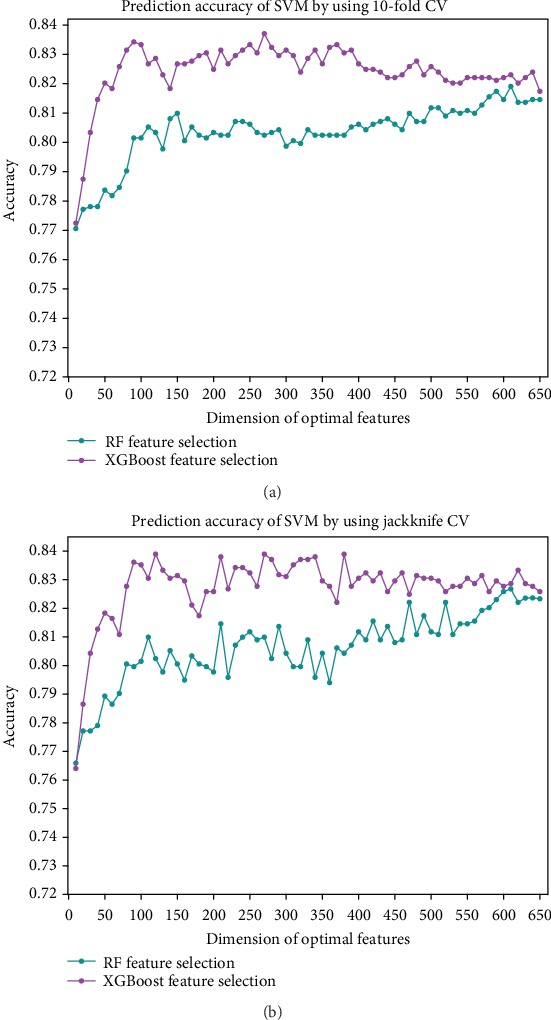
This illustrates how different feature subsets affect the accuracies by using two different feature selection methods. (a) The prediction accuracy of SVM based on the 10-fold CV test. (b) The prediction accuracy of SVM based on the jackknife CV test.

By combining AAC+ACT+CCT features, we firstly obtain a 2020-dimensional vector for each protein. Then, these features are ranked according to their importance by applying RF and XGBoost techniques, respectively. To further determine the optimal feature subset, we calculate the accuracies for top *K* features by using the 10-fold CV and the jackknife CV, respectively, where *K* = 10, 20, 30,…, 650. The results on the PDB1075 dataset are illustrated in Figure 3. As can be observed from Figure 3(a), feature subsets ranked by the XGBoost method could obtain higher accuracies compared with the RF feature ranking technique. When *K* = 270, the highest accuracy of 0.8371 is achieved by using the 10-fold CV. Considering that Figure 3(b) also shows similar results, it is appropriate to pick the top 270 ranked features for the following analyses.

**Figure 4:**
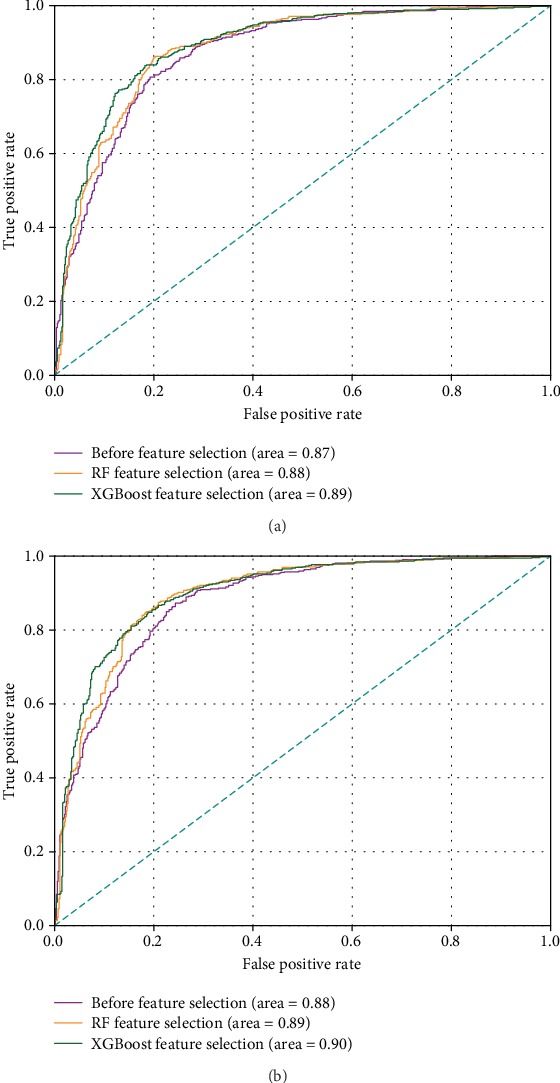
ROC curves of the SVM classifier before and after feature selection. (a) ROC curves based on the 10-fold CV. (b) ROC curves based on the jackknife CV.

Table 3 further examines the effectiveness of the feature selection by comparing the prediction performance of the case without using feature selection, the case using RF feature ranking, and the case using XGBoost feature ranking. Two CV methods, i.e., 10-fold and jackknife, are tested on the PDB1075 dataset by running the SVM classifier, respectively. From Table 3, two main results emerge: (i) the feature selection technique can indeed help to effectively improve the performance of DBPs prediction; and (ii) the XGBoost algorithm may be able to provide better feature ranking than the RF method. We also plot the ROC curves for these experiments in Figure 4, which demonstrates the remarkably consistent findings.

### 3.4. Comparison with Existing Predictors

To objectively evaluate the effectiveness of the proposed method, we make comparisons with some existing predictors on the same datasets. These methods include DNAbinder [24], DNA-Prot [8], iDNA-Prot [10], iDNA-Prot|dis [22], Kmer1+ACC [52], iDNAPro-PseAAC [27], PseDNA-Pro [19], Local-DPP [9], and HMMBinder [29]. The results of jackknife tests on the PDB1075 dataset are listed in Table 4. In addition, Table 5 illustrates five performance measures of various algorithms tested on the PDB186 independent dataset.

As shown in Table 4, the proposed method achieves the values of “ACC” (83.90%), “SP” (83.82%), “MCC” (0.68), and “AUC” (0.9018), which rank second on the benchmark dataset and are merely below those of HMMBinder. The Local-DPP algorithm, which explored local evolutionary information from the PSSM profile, gets the comparable SN of 84% to our method. This indicates that the PSSM profile indeed can provide important clues for predicting DBPs. It is worth mentioning that the Kmer1+ACC method applied the same strategy to extract AAC, ACT, and CCT features from the PSSM profile instead of the HMM profile. Judging from the results of performance comparison, the HMM profile could serve as a better source of information for the identification of DBPs. From the values reported in Table 5, the proposed method obtains the highest ACC, SN, MCC, and AUC among these methods by using the independent dataset test. It should be noted that the HMMBinder method could not provide desired optimal results on the testing set despite achieving the best SP value. This might lead us to believe that there is a risk of overfitting in the HMMBinder method.

In summary, the proposed method shows substantial improvements for identifying DBPs particularly on the independent test, which are attributed to the powerful feature fusion method from the HMM profile and the efficient feature selection by using the XGBoost technique.

## 4. Conclusion

In this paper, we propose a method called HMMPred, which makes an effective improvement on the existing HMM profile-based method to predict DBPs by integrating three feature extraction techniques (i.e., AAC, ACT, and CCT) and adding the application of a prominent feature selection method called XGBoost. Then, the top 270-dimensional features are fed into the SVM classifier to train the model. Based on the comprehensive assessment, using the 10-fold CV, the jackknife CV, and the independent test, it is noteworthy that our method performs well compared to other existing methods and even achieves superior performance on the independent test. In our future work, we would like to develop a web server for the public use and continue to enhance the existing methods for achieving more precise identification of DBPs.

## Figures and Tables

**Table 1:** Prediction results of SVM and RF classifiers based on the 10-fold CV.

Classifier	Feature extraction method	ACC	SN	SP	MCC	AUC
SVM	AAC	0.7893	0.8224	0.7582	0.5810	0.8586
	ACT	0.7004	0.6795	0.7200	0.3999	0.7492
	CCT	0.7678	0.7336	0.8000	0.5352	0.8309
	AAC+ACT+CCT	0.8034	0.8147	0.7927	0.6071	0.8717

RF	AAC	0.7772	0.8147	0.7418	0.5571	0.8600
	ACT	0.7369	0.7394	0.7345	0.4737	0.8022
	CCT	0.7566	0.7896	0.7255	0.5154	0.8232
	AAC+ACT+CCT	0.7781	0.8205	0.7382	0.5596	0.8437

**Table 2:** Prediction results of SVM and RF classifiers based on the jackknife CV.

Classifier	Feature extraction method	ACC	SN	SP	MCC	AUC
SVM	AAC	0.7912	0.8185	0.7655	0.5841	0.8663
	ACT	0.7004	0.6795	0.7200	0.3999	0.7641
	CCT	0.7650	0.7297	0.7982	0.5296	0.8373
	AAC+ACT+CCT	0.8015	0.8127	0.7909	0.6034	0.8806

RF	AAC	0.7930	0.8161	0.7618	0.5885	0.8705
	ACT	0.7369	0.7413	0.7327	0.4738	0.8125
	CCT	0.7547	0.7761	0.7345	0.5106	0.8299
	AAC+ACT+CCT	0.7706	0.8050	0.7382	0.5437	0.8539

**Table 3:** Performance comparison before and after feature selection.

Feature selection	CV methods	ACC	SN	SP	MCC	AUC
Before	10-fold	0.8034	0.8147	0.7927	0.6071	0.8720
	Jackknife	0.8015	0.8127	0.7909	0.6034	0.8805
RF	10-fold	0.8221	0.8243	0.8200	0.6441	0.8819
	Jackknife	0.8267	0.8262	0.8272	0.6533	0.8946
XGBoost	10-fold	0.8371	0.8301	0.8436	0.6738	0.8896
	Jackknife	0.8390	0.8398	0.8382	0.6778	0.9018

**Table 4:** Performance comparison on the PDB1075 dataset.

Methods	ACC	SN	SP	MCC	AUC
DNAbinder	0.7395	0.6857	0.7909	0.48	0.8140
DNA-Prot	0.7255	0.8267	0.5976	0.44	0.7890
iDNA-Prot	0.7540	0.8381	0.6473	0.50	0.7610
iDNA-Prot|dis	0.7730	0.7940	0.7527	0.54	0.8260
Kmer1+ACC	0.7523	0.7676	0.7376	0.50	0.8280
iDNAPro-PseAAC	0.7656	0.7562	0.7745	0.53	0.8392
PseDNA-Pro	0.7655	0.7961	0.7363	0.53	—
Local-DPP	0.7920	0.8400	0.7450	0.59	—
HMMBinder	0.8633	0.8707	0.8555	0.72	0.9026
Our method	0.8390	0.8398	0.8382	0.68	0.9018

**Table 5:** Performance comparison on the independent dataset.

Methods	ACC	SN	SP	MCC	AUC
DNAbinder	0.6080	0.5700	0.6450	0.216	0.6070
DNA-Prot	0.6180	0.6990	0.5380	0.240	—
iDNA-Prot	0.6720	0.6770	0.6670	0.344	—
iDNA-Prot|dis	0.7200	0.7950	0.6450	0.445	0.7860
Kmer1+ACC	0.7096	0.8279	0.5913	0.431	0.7520
iDNAPro-PseAAC	0.7150	0.8276	0.6022	0.442	0.7780
Local-DPP	0.7900	0.9250	0.6560	0.625	—
HMMBinder	0.6902	0.6153	0.7634	0.394	0.6324
Our method	0.8118	0.9462	0.6774	0.648	0.8715

## Data Availability

The datasets and source codes for this study are freely available to the academic community at: https://github.com/taigangliu/HMMPred.
